# Pro-inflammatory diet, cardio-metabolic risk factors and risk of type 2 diabetes: A cross-sectional analysis using data from RaNCD cohort study

**DOI:** 10.1186/s12872-022-03023-8

**Published:** 2023-01-07

**Authors:** Nazli Namazi, Javad Anjom-Shoae, Farid Najafi, Mohammad Hossein Ayati, Mitra Darbandi, Yahya Pasdar

**Affiliations:** 1 Diabetes Research Center, Endocrinology and Metabolism Clinical Sciences Institute, Tehran University of Medical Sciences, Tehran, Iran; 2 Faculty of Nutritional Sciences, Tehran University of Medical Sciences, Tehran, Iran; 3 Research Center for Environmental Determinants of Health (RCEDH), Health Institute, Kermanshah University of Medical Sciences, Kermanshah, Iran; 4 School of Traditional Medicine, Tehran University of Medical Sciences, Tehran, Iran; 5 Research Center for Integrative Medicine in Aging, Aging Research Institute, Tabriz University of Medical Sciences, Tabriz, Iran

**Keywords:** Dietary inflammatory index, Type 2 diabetes mellitus, Cardiometabolic, Dietary pattern, PERSIAN cohort

## Abstract

**Background:**

Inflammation and cardiometabolic risk factors can be involved in developing type 2 diabetes mellitus (T2DM). This study aimed to investigate and compare the association between a pro-inflammatory diet and cardiometabolic risk factors in patients with T2DM and non-T2DM cases.

**Methods:**

In this cross-sectional population-based study, considering the baseline data of the Ravansar Non-Communicable Disease (RaNCD) cohort, patients with T2DM (*n* = 785) and non-T2DM cases (*n* = 8254) were included. The dietary inflammatory index (DII) was calculated using a food frequency questionnaire (FFQ) and was classified into four groups (quartiles) with lowest to highest scores. Logistic regression analysis was conducted to determine the association between DII and cardiometabolic risk factors in both groups.

**Results:**

The participants were 9,039 (4140 men and 4889 women) with a mean age of 47.4 ± 8.2 years; the mean body mass index (BMI) and DII were 27.49 ± 4.63 kg/m^2^ and − 2.49 ± 1.59, respectively. After adjustment for confounding factors, we found that DII can increase the risk of T2DM by 61% (95% CI 1.27 to 2.05, *P* < 0.001). A comparison of two groups revealed that the association of DII, obesity/overweight and dyslipidemia were also significant in both diabetic (*P* < 0.05) and non-diabetic cases (*P* < 0.05). However, no significant association was found between DII, MetS, and hypertension in either of the groups. The association between DII and cardiovascular diseases (CVDs) was only significant in diabetic patients (1.65; 95%CI: 1.02 to 2.65, *P* = 0.04) and T2DM showed an interaction with the association between DII and CVDs.

**Conclusion:**

Inflammatory potential of diet may increase the risk of T2DM. Although it can increase the risk of some cardiometabolic risk factors in both diabetic and non-diabetic cases, its effects were greater among patients with T2DM. However, further prospective studies are required to confirm these associations.

## Introduction

Dietary patterns are one of the main determinants of chronic systemic inflammation which can be resulted in an increasing level of pro-inflammatory cytokines including tumour necrosis factor α (TNF-α), high sensitivity C-reactive protein (hs-CRP), and interleukin 6 (IL-6) [1–3]. The Western dietary pattern, characterized by high consumption of high-fat dairy products, refined grains as well as red and processed meat, has been associated with a higher level of inflammation and an increased risk of inflammation-related diseases [4, 5]. However, a diet rich in whole grains, fruits and green vegetables, such as the Mediterranean and Dietary Approaches to Stop Hypertension (DASH) diet, has been linked with lower concentrations of inflammation markers [6, 7]. Such healthy dietary patterns contain anti-inflammatory nutrients including vitamin C, beta-carotene, n-3 PUFAs, and dietary fibers, which were shown to induce a balance between anti-inflammatory and pro-inflammatory parameters and lower the risks of various chronic diseases [8].

Dietary Inflammatory Index (DII) has been developed, as a population-based dietary score, to specifically reflect the inflammatory potential of dietary factors and to classify the individuals’ diets into the most pro- and anti-inflammatory ones. This index measures the effects of diet on various inflammatory biomarkers by considering the pro- or anti-inflammatory properties of its components, including different macro- and micronutrients and specific food items [9]. An inverse association was reported between DII and other indices which showed healthy dietary patterns such as healthy eating index (HEI) and alternate healthy eating index (AHEI). It is also suggested that DII can be considered a useful tool to examine the healthy eating of adults [10].

Prior evidence also is accumulating on the role of DII in different chronic diseases, which indicated that DII scores are associated with an increased risk of CVDs, obesity, and T2DM [11]. However, there are limited population-based studies, particularly on diabetes. Asadi et al. reported that there was no significant link between the DII and CVDs, stable angina, and myocardial infarction in Mashhad population [12]. In another study by Denova-Gutiérrez et al., the risk of T2DM was 3 times greater in a Mexican population with high scores of DII, compared with those with the lowest scores [13]. Laouali et al. also found that a higher DII score is associated with a higher risk (23%) of T2DM in French adults [14].

Despite the growing interest in the link between inflammatory biomarkers and inflammation-related chronic diseases, the association of the DII and intermediate risk factors of cardiometabolic disorders is insufficiently investigated, particularly in Iranian patients with T2DM. This is particularly important due to differences in dietary habits, genetics, and race and their interactions in various populations, thus, examining the association between DII and cardiometabolic risk factors is likely to be different. Accordingly, the current study aimed to explore and compare the association of DII with cardiometabolic risk factors in both diabetic and non-diabetic populations.

## Methods

### Study design

This cross-sectional study was designed according to the baseline data of the Ravansar Non-Communicable Disease (RaNCD) cohort study, which was conducted in Ravansar, Iran [15]. RaNCD study is a part of the Prospective Epidemiological Research Studies in IRAN (PERSIAN) cohort study, carried out in different cities with the coordination of the Ministry of Health and Medical Education in Iran [16]. Further details of the RaNCD study have been explained elsewhere [15].

### Inclusion and exclusion criteria

**Fig. 1:**
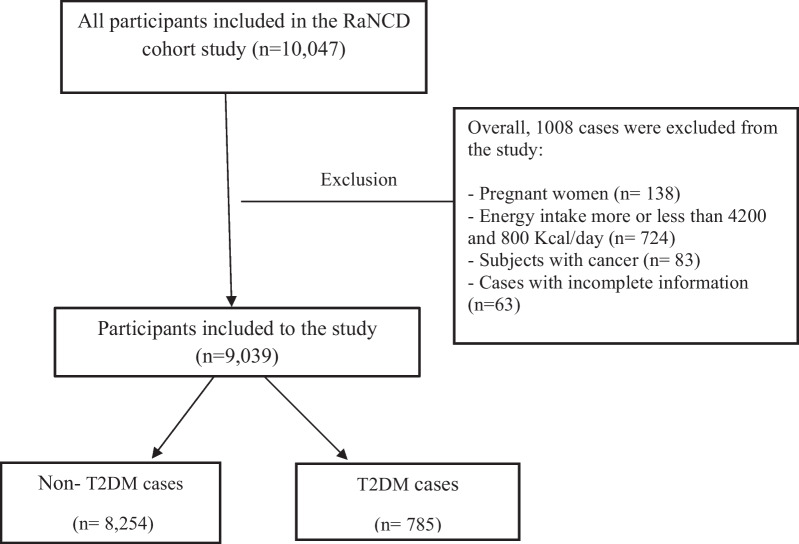
Flow chart of the study

Participants who met the following criteria were included: (i) capacity to cooperate with the project team, (ii) age range between 35 and 65 years old, (iii) being the residence of Ravansar for at least 1 year, with a minimum of nine months per year being in the city, and the possibility of staying in Ravansar during the next years, and (iv) having Iranian nationality. Of 10,047 participants in the RaNCD cohort study, 9039 men and women including patients with T2DM (n = 785), and non-diabetic ones (*n* = 8254) were included. The flowchart of sampling is provided in Fig. 1.

### Ethics approval

All eligible individuals signed written informed consent at baseline. The present study was conducted in accordance with the Declaration of Helsinki and was registered with the Department of Research and Technology after getting approval from the Ethics Committee of Kermanshah University of Medical Sciences (KUMS.REC.1394.318).

### Data collection and measurements

All details of data collection and assessments are available in the RaNCD protocol [15]. Briefly, demographic characteristics and other information were assessed face-to-face using the digital questionnaire. The socio-economic status (SES) was created, using 18 items (including education level, residence place housing and welfare amenities) by principal component analysis (PCA) method. Finally, the SES was categorized from the lowest to the highest in three groups. Individuals, who never smoked, were defined as non-smokers and current smokers were those who reported smoking a minimum of 100 cigarettes. And, former smokers were those who had given up smoking with a history of at least 100 cigarettes throughout their lifetime [17]. Physical activity was assessed using the standard RaNCD cohort questionnaire [22 questions] and was classified into three levels: low, medium and high according to MET/hour per day [18]. We used the BSM 370 (Biospace Co, Seoul, Korea) to measure the height (precision: 0.1 cm) and Bio-Impedance Analyzer BIA (Inbody 770, Inbody Co, Seoul, Korea) to measure the body weight (precision: 0.5 kg). Other anthropometric indices were also measured including BMI, visceral fat area (VFA), Waist to Hip Ratio (WtHR) and Waist Circumference (WC).

Visceral adipose index (VAI) was calculated based on specific formulas for men and women [19] by the following formula:

$${\text{Males}}:{\text{VAI}} = \left( {\frac{{{\text{WC}} ({\text{cm}})}}{{39.68 + \left( {1.88 \times {\text{BMI}} \left( {{\text{kg/m}}^{2} } \right)} \right)}}} \right) \times \left( {\frac{{{\text{TG}} \left( {{\text{mmol}}/{\text{l}}} \right)}}{1.03}} \right) \times \left( {\frac{1.31}{{{\text{HDL}}\left( {{\text{mmol}}/{\text{l}}} \right)}}} \right)$$

$${\text{Females}}:{\text{VAI}} = \left( {\frac{{\text{WC(cm)}}}{{39.58 + \left( {1.89 \times {\text{BMI}} \left( {{\text{kg}}/{\text{m}}^{2} } \right)} \right)}}} \right) \times \left( {\frac{{{\text{TG}} \left( {{\text{mmol}}/{\text{l}}} \right)}}{0.81}} \right) \times \left( {\frac{1.52}{{{\text{HDL}}\left( {{\text{mmol}}/{\text{l}}} \right)}}} \right)$$

Biochemical measurements were also done by collecting participants’ blood samples. We asked them to be fasted overnight for at least 8–12 h. Glucose levels and lipid profile were measured by enzymatic methods after sample collections After taking the blood samples, we kept the serum samples in a freezer at − 72 °C for further measurements.

### Assessment of dietary intake

A 118-item food frequency questionnaire (FFQ) was used to examine dietary intake. The validity of this questionnaire was examined earlier [20]. Details of the assessment were explained elsewhere [15]. Briefly, a face-to-face interview was conducted to fill out the questionnaire. Frequency and the amount of food items consumed, including local foods, during the past year were asked to estimate the participants’ dietary intake.

### Assessment of DII

Using FFQ, DII was calculated. Shivappa et al. found that 45 foods items were linked with at least one of the inflammatory parameters such as C-reactive protein (CRP), Interleukin- 6 (IL-6), Interleukin-1b (IL-1b), Tumor Necrosis Factor-a (TNF-a) or anti-inflammatory markers including Interleukin-4 (IL-4) and Interleukin-10 (IL-10) [9, 21].

The method for calculation of DII has been published in the previous study [9, 21]. Briefly, the DII score was obtained using 31 food items (out of 45 items introduced for DII), extracted from FFQ by Shivappa criteria [9]. Food items with anti-inflammatory characteristics obtained a score of + 1, and pro-inflammatory ones received a score of − 1. The scores for those with no effect on the levels of inflammatory markers were 0. The greater positive DII scores show more pro-inflammatory diets, and higher negative scores point to a more anti-inflammatory dietary pattern. Generally, the total score for DII can be ranged between − 8.87 (the greatest anti-inflammatory score) and + 7.98 (the highest pro-inflammatory score).

### Definitions of T2DM, CVDs, metabolic syndrome (MetS) and other metabolic disorders

Fasting blood sugar (FBS) levels equal to or higher than 126 mg/dL and/or treatment with anti-diabetic medications were considered for the diagnosis of T2DM. Participants with a history of and/or treatment for one or more types of heart diseases such as stroke, myocardial infarction (MI), coronary artery disease, and/or taking CVDs medications were categorized as patients with CVDs. Participants with systolic blood pressure (SBP) of equal to or greater than 140 mmHg and/or diastolic blood pressure (DBP) of equal to or greater than 90 mmHg and/or those with a current use of antihypertensive drugs were classified as patients with hypertension [22]. Dyslipidemia was also defined as serum levels of TC ≥ 240 mg/dl and/or LDL-C ≥ 160 mg/dL and/or TG ≥ 200 mg/dL and/or HDL- C < 40 mg/dL or a history of taking medications for dyslipidemia [23]. Subjects with BMI equal to or greater than 25 kg/m2 were also considered as subjects with overweight/obesity. Moreover, International Diabetes Federation (IDF) criteria were also considered to identify subjects with the MetS [21, 24].

### Statistical analysis

Personal characteristics and other assessments across the quartiles of the DII score were reported as Mean ± Standard Deviation (SD) for continuous variables, and qualitative variables were provided by percentages. The normality of the data was checked using the Kolmogorov–Smirnov test. Comparison of baseline characteristics between the two groups of with and without T2DM was performed using t-test and chi-square test. To compare DII quartiles, the one-way ANOVA test was applied. Crude and adjusted logistic regression models were applied to assess the association (odd ratio) between DII quartiles and cardiometabolic risk factors with odds of T2DM. Estimates with a 95% confidence interval and a P-value lower than 0.05 were reported as significant. In the adjusted model, age, sex, energy intake, physical activity, smoking, and SES were taken into the account. All analyses in the current study were performed using Stata version 14.2 software (Stata Corp, College Station, TX, USA).

## Results

### Characteristics of the study population

Out of 10,047 participants in the RaNCD cohort study, 9,039 individuals including T2DM (n = 785) and non-T2DM cases (n = 8254) were considered for the present study. Baseline characteristics of participants are presented in Table 1. Mean age of the study population was 47.4 ± 8.2 years old and around 46% of them were men with or without diabetes. There were significant differences in terms of smoking, sleep duration, and physical activity levels between diabetic and non-diabetic cases (P-values for all < 0.05). However, no considerable differences were found in socioeconomic status (SES) between the two groups (*P* = 0.06).

**Table 1:** Baseline characteristics of the study population (*N* = 9039)

Parameters	Total (*n* = 9039)	Non-T2DM (*n* = 8,254)	T2DM (*n* = 785)	*P* value
Age (year)	47.48 ± 8.29	47.07 ± 8.23	51.70 ± 7.53	< 0.001
Gender, n (%)				
Male	4140 (45.80)	3804 (46.09)	336 (42.80)	0.078
Female	4889 (54.20)	4450 (53.91)	449 (57.20)	
Smoking, n (%)				
Never	7234 (80.57)	6627 (80.85)	607 (77.62)	< 0.001
Current smoker	992 (11.05)	920 (11.22)	72 (9.21)	
Former smoker	753 (8.39)	650 (7.93)	103 (13.17)	
Physical activity, (Met hour per week), n (%)				
Low	2784 (30.80)	2504 (30.34)	280 (35.67)	< 0.001
Moderate	4356 (48.19)	3966 (47.05)	390 (49.68)	
Vigorous	1899 (21.01)	1784 (21.61)	115 (14.65)	
Socioeconomic status, n (%)				
Low	3024 (33.47)	2777 (33.66)	247 (31.51)	0.062
Moderate	3000 (33.20)	2710 (32.84)	290 (36.99)	
High	3011 (33.33)	2764 (33.50)	247 (31.51)	
DII score, Mean ± SD	-2.49 ± 1.59	− 2.50 ± 1.59	− 2.37 ± 1.59	0.022
Energy intake (kcal/day)	2492.71 ± 736.47	2503.66 ± 733.45	2377.48 ± 758.46	0.001
Carbohydrate (%E)	61.43 ± 6.20	61.51 ± 6.18	60.51 ± 6.35	< 0.001
Lipid (%E)	26.81 ± 5.94	26.79 ± 5.93	27.06 ± 5.99	0.201
Protein (%E)	13.75 ± 4.93	13.67 ± 2.16	14.56 ± 2.28	< 0.001
Sleep duration (hour/24)	7.10 ± 1.23	7.11 ± 1.22	6.70 ± 1.32	0.001
BMI (kg/m^2^)	27.49 ± 4.63	27.35 ± 4.31	28.93 ± 4.38	< 0.001
WHR	0.94 ± 0.06	0.94 ± 0.06	0.96 ± 0.06	< 0.001
WC (cm)	97.28 ± 10.50	96.93 ± 10.50	100.86 ± 9.82	< 0.001
VFA (cm^2^)	122.88 ± 51.53	121.25 ± 51.43	14,014 ± 49.42	< 0.001
VAI (male)	0.05 ± 0.06	0.05 ± 0.06	0.07 ± 0.08	< 0.001
VAI (female)	0.07 ± 0.09	0.07 ± 0.08	0.11 ± 0.12	< 0.001
TG (mg/dl)	137.10 ± 82.64	132.96 ± 77.01	180.01 ± 119.75	< 0.001
HDL-C (mg/dl)	46.53 ± 11.31	46.75 ± 11.33	44.14 ± 11.06	< 0.001
LDL-C (mg/dl)	102.01 ± 25.41	101.85 ± 25.12	103.68 ± 28.17	0.055
T-C (mg/dl)	185.25 ± 37.83	184.77 ± 37.18	190.34 ± 43.86	< 0.001
TG/ HDL (mg/dl)	3.29 ± 2.66	3.17 ± 2.48	4.54 ± 3.89	< 0.001
LDL/HDL (mg/dl)	2.29 ± 0.70	2.28 ± 0.69	2.44 ± 0.73	< 0.001
T-C/HDL (mg/dl)	4.16 ± 1.11	4.13 ± 1.10	4.49 ± 1.26	< 0.001
Anti-diabetic medications (%)	519 (85.41)	0 (0)	519 (84.53)	
Hypertension, n (%)	1438 (15.91)	1181 (14.31)	257 (32.74)	0.012
Dyslipidemia, n (%)	3961 (43.82)	3444 (41.73)	517 (65.86)	< 0.001
CVDs, n (%)	1565 (17.31)	1227 (14.87)	338 (43.06)	< 0.001
Anti-CVDs medications (%)	1422 (39.98)	1102 (37.44)	320 (52.12)	< 0.001
Anti-Hypertension medications (%)	979 (23.74)	784 (22.52)	195 (30.33)	< 0.001
Anti-Dyslipidemia medications (%)	401 (11.27)	222 (7.54)	179 (29.15)	< 0.001

*BMI* body mass index, *DII* dietary inflammatory index, *WHR* waist-to-hip ratio, *WC* waist circumference, *VFA* visceral fat area, *SLM* skeletal lean mass, *TG* triglycerides, *HDL-C* high-density lipoprotein cholesterol, *LDL-C* low-density lipoprotein cholesterol, *T-C* total cholesterol, *CVDs* cardiovascular diseases, *VAI* visceral adiposity index

The total mean DII score was − 2.49 ± 1.59. As indicated in Table 1, this score among non-T2DM cases was significantly fewer (with more anti-inflammatory levels) than those with T2DM (mean − 2.50 vs. − 2.37, *P* = 0.022). Regarding anthropometric indices, significant differences were found between the two groups. BMI, WC, WHR as well as VAI in patients with T2DM were greater than in non-diabetic ones (p-values < 0.01 for all). Moreover, percentages of subjects with metabolic disorders such as hypertension, dyslipidemia, and CVDs in diabetic patients were higher than non-T2DM ones (P-values < 0.01 for all), as expected.

### Characteristics of participants in DII categories

In Table 2, the characteristics of all participants, regardless of the diabetic status of participants, are indicated. Based on the findings, mean DII ranged from − 4 to − 0.1 (Quartile 1 to Quartile 4) and most individuals (*n* = 2391) adhered to anti-inflammatory diets. In addition, most percentage (about 35%) of subjects with low SES had the most anti-inflammatory diet. Regarding the level of physical activity, no significant differences were found between the quartiles of DII (*P* = 0.13).

**Table 2:** Characteristics of participants according to quartiles of the dietary inflammatory index score

Variable	Dietary inflammatory index (DII)				*P* value
	Quartile 1: Most Anti-Inflammatory	Quartile 2	Quartile 3	Quartile 4: Most Pro-Inflammatory	
Frequency, n	2,391	2,382	2,337	1,929	–
DII, mean ± SD	− 4.03 ± 0.41	− 3.12 ± 0.24	− 2.10 ± 0.38	− 0.10 ± 1.05	–
Age, mean ± SD	48.65 ± 8.46	47.62 ± 8.33	46.90 ± 8.16	46.54 ± 7.90	
Gender, n (%)					
Male	964 (23.29)	1051 (25.39)	1109 (26.79)	1016 (24.54)	< 0.001
Female	1427 (29.13)	1331 (27.17)	1228 (25.07)	913 (18.64)	
Smoking, n (%)					
Never	1905 (26.33)	1921 (26.56)	1871 (25.86)	1537 (21.25)	0.310
Current smoker	261 (26.31)	276 (27.82)	249 (25.10)	206 (20.77)	
Former smoker	213 (28.29)	168 (22.31)	200 (26.56)	172 (22.84)	
Socio-economic status, n (%)					
Low	1132 (37.43)	815 (26.95)	593 (19.61)	484 (16.01)	< 0.001
Moderate	671 (22.37)	820 (27.33)	842 (28.07)	667 (22.23)	
Vigorous	587 (19.50)	746 (24.78)	902 (29.96)	776 (25.77)	
Physical activity (Met-h/week), n (%)					
Light	707 (25.40)	776 (27.87)	723 (25.97)	578 (20.76)	0.133
Moderate	1183 (27.16)	1097 (25.18)	1163 (26.70)	913 (20.96)	
High	501 (26.38)	509 (26.80)	451 (23.75)	438 (23.06)	
Hypertension, n (%)	431 (29.97)	352 (24.48)	353 (24.55)	302 (21.00)	0.009
Dyslipidemia, n (%)	985 (24.87)	1006 (25.40)	1027 (25.93)	943 (23.81)	< 0.001
CVDs, n (%)	459 (29.33)	406 (25.94)	405 (25.88)	295 (18.85)	< 0.001
T2DM, n (%)	194 (24.71)	193 (24.59)	202 (25.73)	196 (24.97)	0.017
Insulin (yes/no) (%)	6 (20.69)	5 (17.24)	8 (27.59)	10 (34.48)	0.299
Anti-diabetic medications (%)	122 (23.51)	122 (23.51)	142 (27.36)	133 (25.63)	< 0.001
Anti-CVDs medications (%)	425 (29.89)	369 (25.95)	359 (25.25)	269 (18.92)	0.489
Anti-Hypertension medications (%)	269 (30.23)	257 (26.25)	240 (24.51)	186 (19.00)	0.433
Anti-Dyslipidemia medications (%)	93 (23.19)	100 (24.94)	101 (25.19)	107 (26.68)	0.001
BMI (kg/m^2^), mean ± SD	26.99 ± 4.68	27.41 ± 4.62	27.58 ± 4.60	27.96 ± 4.56	< 0.001
WHR, mean ± SD	0.93 ± 0.06	0.94 ± 0.06	0.94 ± 0.06	0.95 ± 0.06	< 0.001
WC (cm)	97.17 ± 10.64	97.46 ± 10.48	97.35 ± 10.44	97.08 ± 10.44	0.644
VFA (cm^2^)	119.70 ± 50.81	123.29 ± 51.84	123.90 ± 51.53	125.08 ± 51.90	0.003
VAI (male)	0.04 ± 0.05	0.05 ± 0.07	0.05 ± 0.06	0.05 ± 0.06	< 0.001
VAI (female)	0.07 ± 0.07	0.07 ± 0.10	0.07 ± 0.08	0.09 ± 0.09	< 0.001
Sleep duration(hour/24)	7.14 ± 1.22	7.12 ± 1.24	7.06 ± 1.21	7.07 ± 1.19	0.097

There were significant differences in BMI, WHR and VFA across quartiles of DII score. Compared with those in the lowest quartile, participants in the highest quartile of DII score had greater BMI (*P* < 0.001), WHR (*P* < 0.001), and VFA (*P* = 0.003). Regarding their dietary intake, subjects with the most pro-inflammatory diets consumed greater total energy (*P* < 0.001) compared to those with the most anti-inflammatory diet (Table 3). The intake of all nutrients and food groups except carbohydrates (*P* = 0.98) and fat (*P* = 0.12) were significantly different across the categories of the DII score (Table 3).

**Table 3:** Description of food parameters participants according to the dietary inflammatory index score

Food parameters	Quartile 1: Most Anti-Inflammatory	Quartile 2	Quartile 3	Quartile 4: Most Pro-Inflammatory	*P* value
Energy intake (kcal/d)	2136.31 ± 661.18	2332.87 ± 651.96	2622.40 ± 690.17	2974.70 ± 675.82	< 0.001
Carbohydrate intake (%E)	61.42 ± 6.64	61.50 ± 6.16	61.39 ± 6.01	61.39 ± 5.97	0.980
Protein intake (%E)	13.16 ± 2.01	13.53 ± 2.10	13.85 ± 2.12	14.60 ± 2.34	< 0.001
Fat intake (%E)	26.62 ± 6.40	26.73 ± 5.99	26.98 ± 5.74	26.92 ± 5.60	0.123
Saturated fat (g/d)	25.32 ± 12.80	26.57 ± 11.95	29.77 ± 12.68	32.96 ± 12.66	< 0.001
Monounsaturated fats (g/d)	15.99 ± 7.80	18.13 ± 7.64	20.95 ± 8.65	24.22 ± 9.06	< 0.001
Polyunsaturated fats (g/d)	7.99 ± 4.04	9.94 ± 4.21	12.11 ± 5.25	14.66 ± 5.57	< 0.001
Trans fat (g/d)	0.16 ± 0.23	0.24 ± 0.27	0.32 ± 0.35	0.42 ± 0.43	< 0.001
Cholesterol (mg/d)	223.32 ± 126.73	252.63 ± 125.50	293.64 ± 147.27	347.93 ± 156.48	< 0.001
Red meat (g/d)	19.73 ± 28.58	17.80 ± 24.19	22.37 ± 27.75	23.57 ± 29.15	< 0.001
Poultry (g/d)	31.38 ± 26.48	40.81 ± 32.39	47.15 ± 39.60	61.14 ± 47.98	< 0.001
Fish (g/d)	3.23 ± 5.60	4.89 ± 7.50	7.31 ± 9.79	10.91 ± 13.40	< 0.001
Vegetables (g/d)	146.99 ± 88.10	216.47 ± 108.76	300.44 ± 132.99	459.16 ± 201.88	< 0.001
Fruits(g/d)	142.49 ± 128.13	199.48 ± 144.56	277.25 ± 184.41	376.10 ± 236.44	< 0.001
Dairy product (g/d)	420.36 ± 426.97	404.77 ± 369.35	464.69 ± 395.38	500.06 ± 434.54	< 0.001
Legumes (g/d)	18.56 ± 14.53	26.65 ± 19.80	36.27 ± 26.74	57.23 ± 42.32	< 0.001
Egg (g/d)	15.23 ± 16.60	19.44 ± 18.20	21.99 ± 19.48	26.00 ± 22.49	< 0.001
Potato (g/d)	32.50 ± 27.56	42.05 ± 34.38	48.61 ± 38.35	60.36 ± 49.56	< 0.001
Refined grains (g/d)	478.57 ± 180.67	495.54 ± 199.87	519.83 ± 210.28	544.68 ± 215.11	< 0.001
Whole grains (g/d)	6.04 ± 7.63	8.24 ± 9.83	10.96 ± 12.73	16.25 ± 17.31	< 0.001
Sweets & desserts (g/d)	49.25 ± 36.46	56.10 ± 38.50	61.19 ± 40.63	65.45 ± 43.29	< 0.001
Tea & coffee (g/d)	688.76 ± 490.21	726.04 ± 511.40	722.35 ± 480.41	753.19 ± 501.23	0.003
Caffeine (mg/d)	139.10 ± 98.20	146.99 ± 102.58	146.86 ± 96.56	153.40 ± 100.86	< 0.001
Alcohol consumption (g/d)	0.02 ± 0.04	0.03 ± 0.06	0.04 ± 0.08	0.05 ± 0.10	< 0.001
Iron (mg/d)	14.60 ± 5.04	15.93 ± 5.55	17.98 ± 5.73	21.28 ± 6.03	< 0.001
Zinc (mg/d)	7.62 ± 2.95	8.61 ± 2.78	10.13 ± 3.20	12.18 ± 3.44	< 0.001
Folate (mcg/d)	459.92 ± 162.29	499.50 ± 181.77	558.52 ± 184.46	668.11 ± 190.01	< 0.001
Vitamin A (mcg/d)	460.20 ± 262.85	589.10 ± 270.82	796.31 ± 347.10	1157.31 ± 518.76	< 0.001
Vitamin C (mg/d)	63.71 ± 36.76	88.58 ± 40.61	119.46 ± 50.42	176.93 ± 75.11	< 0.001
Vitamin D (mcg/d)	0.85 ± 0.60	1.10 ± 0.67	1.36 ± 0.83	1.73 ± 1.02	< 0.001
Vitamin E (mg/d)	5.17 ± 2.38	6.56 ± 253	8.24 ± 3.51	10.56 ± 3.65	< 0.001
Omega − 3 fatty acids (g/d)	0.03 ± 0.02	0.04 ± 0.02	0.05 ± 0.03	0.06 ± 0.04	< 0.001
Omega − 6 fatty acids (g/d)	3.19 ± 2.60	4.11 ± 2.76	5.12 ± 3.21	6.17 ± 3.60	< 0.001

### The association between DII and the risk of T2DM

Logistic regression analysis revealed that the risk of T2DM in subjects who consumed the most pro-inflammatory diet was 28% (95% CI: (1.03 to 1.57)) greater than in those with the most anti-inflammatory ones. After adjustment for confounding factors including age, sex, energy intake, BMI, physical activity, and dyslipidemia, the risk reached 61% (95% CI 1.27–2.05).

### The association between DII and cardiometabolic risk factors

The risk of cardiometabolic disorders in both crude and adjusted models was compared between patients with T2DM and non-T2DM cases and provided in Table 4. In diabetic individuals, the odds of obesity and overweight in the fourth quartile of DII were significantly higher than in the first quartile (Adjusted OR 2.11; 95% CI 1.18, 3.78, *P* = 0.01), and in non-diabetic individuals, was significantly 52% higher in the fourth quartile than in the first quartile (Adjusted OR: 1.52; 95% CI 1.32, 1.76, *P* < 0.001). Regarding dyslipidemia, the odds in the fourth quartile of DII were significantly 58% higher than the first one among patients with diabetes (Adjusted OR: 1.58: 95% CI 1.01, 2.50, *P* = 0.04), and in non-diabetic cases, was 20% greater in the fourth quartile compared to the reference group (Adjusted OR: 1.20: 95% CI 1.05, 1.38, *P* = 0.007). As indicated in Table 4, the association between MetS and DII was significant only in non-diabetic cases (Adjusted OR: 1.18; 95% CI: 1.01, 1.36, P = 0.02).

**Table 4:** Association between cardio-metabolic risk factors and dietary inflammatory index in populations with and without type 2 diabetes mellitus

Cardio-metabolic risk factors	DII	Crude				Adjusted			
		Non-T2DM		T2DM		Non-T2DM		T2DM	
		OR (95% CI)	*P* value	OR (95% CI)	*P* value	OR (95% CI)	*P* value	OR (95% CI)	*P* value
Obesity & Overweight *	Quartile 1	1	–	1	–	1	–	1	–
	Quartile 2	1.15 (1.01, 1.30)	0.028	1.45 (0.88, 2.40)	0.146	1.12 (0.98, 1.28)	0.077	1.35 (0.80, 2.29)	0.271
	Quartile 3	1.27 (1.12, 1.44)	< 0.001	1.30 (0.80, 2.12)	0.289	1.20 (1.04, 1.37)	0.009	1.26 (0.75, 2.12)	0.375
	Quartile 4	1.51 (1.32, 1.74)	< 0.001	2.27 (1.30, 3.94)	0.004	1.52 (1.32, 1.76)	< 0.001	2.11 (1.18, 3.78)	0.012
Dyslipidemia **	Quartile 1	1	–	1	–	1	–	1	–
	Quartile 2	1.04 (0.92, 1.18)	0.494	1.06 (70., 1.61)	0.779	1.02 (0.90, 1.16)	0.734	1.06 (0.68, 1.63)	0.799
	Quartile 3	1.13 (1.01, 1.28)	0.044	0.95 (0.62, 1.41)	0.751	1.10 (0.95, 1.22)	0.257	1.00 (0.65, 1.55)	0967
	Quartile 4	1.34 (1.18, 1.52)	< 0.001	1.45 (0.94, 2.22)	0.090	1.20 (1.05, 1.38)	0.007	1.58 (1.01, 2.50)	0.049
Hypertension **	Quartile 1	1	–	1	–	1	–	1	–
	Quartile 2	0.77 (0.66, 0.91)	0.003	0.85 (0.56, 1.31)	0.470	0.81 (0.686, 0.98)	0.027	0.91 (0.58, 1.42)	0.670
	Quartile 3	0.77 (0.66, 0.91)	0.003	0.98 (0.65, 1.49)	0.937	0.88 (0.73, 1.06)	0.167	1.10 (0.70, 1.73)	0.661
	Quartile 4	0.81 (0.68, 0.97)	0.022	0.86 (0.56, 1.31)	0.473	0.94 (0.77, 1.14)	0.515	1.04 (0.65, 1.66)	0.853
Metabolic syndrome *	Quartile 1	1	–	1	–	1	–	1	–
	Quartile 2	0.98 (0.87, 1.12)	0.852	0.78 (0.47, 1.31)	0.351	1.04 (0.91, 1.20)	0.512	0.76 (0.44, 1.32)	0.336
	Quartile 3	1.01 (0.89, 1.15)	0.873	0.70 (0.42, 1.14)	0.153	1.10 (0.94, 1.25)	0.222	0.75 (0.44, 1.30)	0.315
	Quartile 4	1.02 (0.89, 1.17)	0.743	0.64 (0.39, 1.10)	0.090	1.18 (1.01, 1.36)	0.028	0.70 (0.40, 1.22)	0.214
CVDs **	Quartile 1	1	–	1	–	1	–	1	–
	Quartile 2	0.84 (0.71, 0.99)	0.036	0.99 (0.66, 1.48)	0.885	0.91 (0.76, 1.10)	0.278	1.11 (0.71, 1.74)	0.638
	Quartile 3	0.85 (0.72, 1.01)	0.062	0.95 (0.64, 1.41)	0.803	1.01 (0.84, 1.21)	0.918	1.18 (0.76, 1.85)	0.460
	Quartile 4	0.67 (0.56. 1.02)	0.060	0.94 (0.63, 1.40)	0.770	0.83 (0.67, 1.00)	0.061	1.30 (0.82, 2.03)	0.273
Waist circumference *	Quartile 1	1	–	1	–	1	–	1	–
	Quartile 2	099 (0.85, 1.15)	0.922	0.73 (0.33, 1.58)	0.426	1.10 (0.93, 1.30)	0.279	0.71 (0.29, 1.69)	0.435
	Quartile 3	0.93 (0.81, 1.08)	0.381	0.41 (0.20, 0.83)	0.013	1.06 (0.90, 1.26)	0.474	0.39 (0.18, 0.89)	0.026
	Quartile 4	0.78 (0.67, 0.91)	0.001	0.45 (0.22, 0.92)	0.030	0.98 (0.82, 1.19)	0.836	0.47 (0.21, 1.08)	0.076

*Adjusted for age, sex, physical activity, SES and smoking

**Adjusted for age, sex, physical activity, smoking, SES and body mass index

**Fig. 2:**
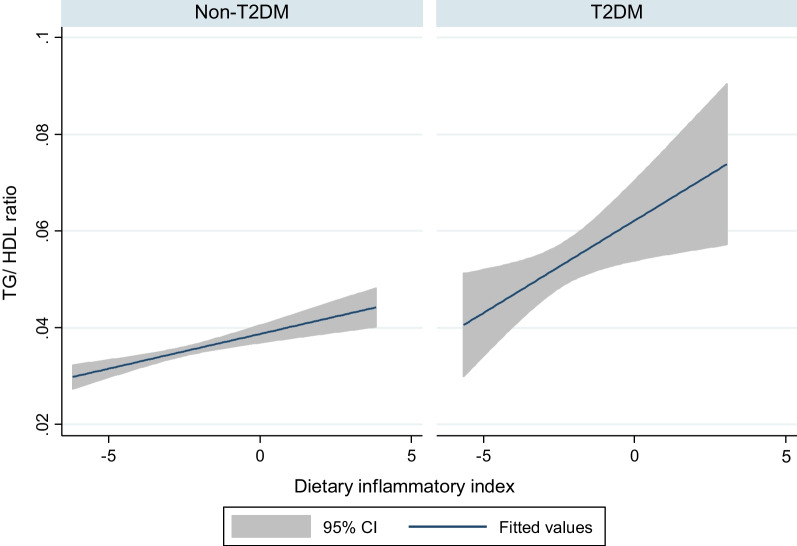
Associations between TG/HDL ratio and dietary inflammatory index in non-T2DM and T2DM participants

The association between DII and an atherogenic index (TG/HDL-C) for both groups is shown in Fig. 2. In both diabetic and non-diabetic cases, direct associations were found between DII and TG/HDL-C. However, the association among diabetic patients was greater than in non-diabetic ones.

## Discussion

In the current population-based study, the possible link between the inflammatory potential of diet and risk of cardiometabolic parameters was investigated in both patients with T2DM and the non-diabetic population. Our results demonstrated that participants with the greatest adherence to a pro-inflammatory diet had a higher risk of T2DM versus those with the lowest adherence, even after adjusting for potentially confounding variables. Comparing diabetic and non-diabetic participants, the DII score was also significantly associated with the risk of various intermediate-risk factors of cardiometabolic disorders including obesity/overweight and dyslipidemia among both T2DM and non-T2DM cases. But regarding MetS, a significant association was observed only in non-diabetic cases. Observing these results among both T2DM and non-T2DM cases may support the roles pro-inflammatory diets can have regardless of the health status of participants. However, it should be considered that these dietary factors’ effects in patients with T2DM were greater than non-diabetic ones.

Overall, previous research has reported that the DII score was associated with several inflammatory processes including obesity [21], CVDs [25] and various cancers [26]. In the current study, we compared DII scores among both healthy and diabetic participants. Interestingly, it has been observed that T2DM patients had more pro-inflammatory dietary intakes than non-T2DM cases, such that, mean DII scores were -2.37 and -2.50 among T2DM and non-T2DM cases, respectively.

As diabetic patients are at a higher risk of CVDs than healthy individuals, it is essential to examine potentially modifiable risk factors for CVDs in such a population and compare them with healthy individuals [4–6]. Dietary indicators are among the most important and potentially modifiable of these risk factors [10–12]. One of the most interesting factors that have received great attention in recent years is the inflammatory potential of diet, which has been quantified by the DII score [18]. Using the DII score to characterize the associations between dietary inflammatory potential and CVDs is of immense importance leading us to conduct food-based novel strategies to prevent and control CVDs. Up to now, there were several studies demonstrating well-established links between DII score and CVDs risk factors [27, 28]. In line with our observations, several, but not all investigations in different populations have reported that a greater pro-inflammatory diet is linked to a higher risk of CVDs risk factors. For instance, in a prospective population-based study among 3726 French participants, a significant association was demonstrated between a higher DII score and an elevated risk of metabolic syndrome, as well as blood pressure, triglycerides which are all among the main risk factors of CVDs [27]. A higher DII score was also reported to be prospectively linked with myocardial infarction (MI) occurrence among the French population [28]. Moreover, no significant relationship was shown between DII score and metabolic syndrome risk in a Mediterranean prospective study in which 6851 participants were followed for 8.3 years [29]. However, Vissers et al. conducted a cohort study on Australian women in the range of 50–55 years and reported no significant relationships between higher DII scores and CVD events including ischemic heart disease, MI, cerebrovascular disease, and Stroke [30]. These contradictory findings could be due to the various characteristics of the examined population such as their age, BMI, socioeconomic status, health status, disease background, race, the mean score of DII and more importantly their dietary habits and lifestyle. Thus, evaluating the links between DII score and various risk factors of CVDs in different population settings is of high importance as these variables have an impact on both DII score and its relationship with cardiovascular health. Another study among 7,216 men (55–80 years old) and women (60–80 years old) conducted by Garcia-Arellano et al. has also reported that a higher DII score was associated with an increased odds of atherosclerosis [31]. Moreover, a large analysis of data obtained in seven countries reported that the DII score was positively correlated with an increased risk of CVDs-related mortality [32]. However, among the prior investigations, only one clinical trial study was carried out on participants with diagnosed diabetes. In this trial which was conducted on 2568 patients, aged 50–75 years, DII score was significantly associated with the CVDs risk factors profile [33]. Our study findings have confirmed these observations and suggested that the inflammatory potential of diet could have detrimental effects even among diabetic patients who have metabolic features associated with insulin resistance. However, insignificant findings in other prior investigations which could be due to different variables including the number of foods used to calculate the DII score, the duration of follow-up, and the definition of cases, provide the necessity that further studies are required in this area to clarify this issue.

The potential mechanisms through which the inflammatory potential of diet might influence CVDs have not been fully elucidated. However, one of the plausible mechanisms could be due to the effects of a pro-inflammatory diet on insulin resistance leading to increased systemic inflammation, which subsequently plays a common pathway for CVDs [34, 35]. Indeed, pro-inflammatory dietary factors increased the expression of some inflammatory cytokines such as IL-1 and TNF-α. These inflammatory markers will in return interfere with insulin signaling and cause insulin resistance [36, 37]. Then, due to the vital roles of insulin in activating nitric oxide, which is a potent vasodilator and antiatherogenic agent, any impairments in insulin signaling such as insulin resistance may lead to hypertension and subsequently CVDs [38, 39]. Cytokines have been also shown to cause attraction and migration of inflammatory cells into vascular tissue and increase the expression of various cell adhesion molecules, which in turn, mediate the adhesion of white blood cells to the vascular endothelium [40]. Moreover, the process of up-regulation of atherogenic gene expression, caused by various dietary inflammatory factors, has been reported to enhance the risks of CVDs [41, 42].

The current study has several limitations that should be addressed. One of the main limitations of the present study was the impossibility of inferring a causal association between DII score and CVDs risk factors due to the observational type of our study. As another limitation of the present study, we were not able to control the confounding effects of genetic and biological differences on the results, since a homogeneous population with a high sample size was used as a control group. Some other confounders, including unmeasured or unknown variables that can affect statistical analysis, have not been controlled in the current study. Although we used a validated FFQ, all 45 items for the DII score were not considered in calculating the total score, and a measurement error was also unavoidable due to using the FFQ for the nutritional assessment of participants. The current study, however, was the only study aimed to determine the association of DII scores with CVDs among a large sample size of Kurdish adults, which could be considered the first strength of our study. High quality of data collection, population-based study, and adjustment for several potential confounders to reach an independent association between DII score and CVDs risk factors are among other strengths of the present study.

## Conclusion

Inflammatory potential of diet can increase the risk of T2DM by 61%. Although it can increase the risk of some cardiometabolic risk factors in both diabetic and non-diabetic cases, its effects in patients with T2DM were greater than in non-diabetic ones. Accordingly, providing healthy dietary recommendations and a personalized diet full of anti-inflammatory food items and the least pro-inflammatory foodstuffs such as Mediterranean and DASH diets can be helpful for patients with T2DM to prevent diabetes complications. However, further studies, with a prospective design, are required to confirm these observations.

## Data Availability

Data are available from the corresponding author upon reasonable request due to privacy or other restrictions.

## Abbreviations

BMI: Body mass index
DII: Dietary inflammatory index
WHR: Waist hip ratio
WC: Waist circumference
VFA: Visceral fat area
TG: Triglycerides
HDL-C: High-density lipoprotein cholesterol
LDL-C: Low-density lipoprotein cholesterol
T-C: Total cholesterol
CVDs: Cardiovascular diseases
VAI: Visceral adiposity index
FBS: Fasting blood sugar
SBP: Systolic blood pressure
DBP: Diastolic blood pressure
RaNCD: Ravansar non-communicable diseases
MI: Myocardial infarction
CRP: C-reactive protein
TNF-α: Tumor necrosis factor alpha, high sensitivity
IL-6: Interleukin 6
IL-4: Interleukin 4
IL-10: Interleukin 10
MI: Myocardial infarction
DASH: Dietary approaches to stop hypertension
HEI: Healthy eating index
AHEI: Alternate healthy eating index
T2DM: Type 2 diabetes mellitus
SES: Socio-economic status
